# Hepatocellular Carcinoma with Both Fibrolamellar and Classical Components: An Unusual Morphological Pattern

**DOI:** 10.1155/2015/609780

**Published:** 2015-02-26

**Authors:** Diana Castro-Villabón, Luis E. Barrera-Herrera, Paula A. Rodríguez-Urrego, Rachel Hudacko, Alonso Vera, Johanna Álvarez, Rafael Andrade, Rocío López

**Affiliations:** ^1^Department of Pathology and Laboratories, Hospital Universitario Fundación Santa Fe de Bogotá, Calle 119 No. 7–75, Bogotá 110111, Colombia; ^2^School of Medicine, University of Los Andes, Carrera 1 No. 18A-12, Bogotá 111711, Colombia; ^3^Department of Pathology, Good Samaritan Regional Medical Center, 255 Lafayette Avenue, Suffern, NY 10901, USA; ^4^Transplant and Hepatobiliary Surgical Service, Hospital Universitario Fundación Santa Fe de Bogotá, Calle 119 No. 7–75, Bogotá 110111, Colombia; ^5^School of Medicine, National University of Colombia, Carrera 45 No. 26-85, Bogotá 111711, Colombia

## Abstract

Fibrolamellar carcinoma (FLC) is an uncommon form of primary liver malignancy with unique clinical, histological, and biological characteristics. It is usually seen in young adults without underlying liver disease. Histologically, it shows large cells with abundant eosinophilic cytoplasm, large vesicular nuclei, prominent nucleoli, and lamellar type fibrosis. In contrast, classical hepatocellular carcinoma (HCC) is typically present in elderly male patients with cirrhosis. It is the most common histological subtype, and it is characterized by its resemblance to the normal liver, both in its growth pattern and its cytology. The unusual case of a liver carcinoma that presented with histological features of both FLC and classical HCC is herein reported. This was the case of a 37-year-old female complaining of diffuse abdominal discomfort and epigastric pain for two months. She was referred to us for further management after she was diagnosed with HCC in a noncirrhotic liver. She underwent a left-sided hepatectomy. A yellow nodular mass with well-defined borders and a necrotic center was present in the resection specimen. The morphological features and immunohistochemical studies were consistent with a diagnosis of FLC mixed with classical HCC. The patient was followed up for five months, and no signs of recurrence were evident.

## 1. Introduction

Fibrolamellar carcinoma (FLC) is a rare variant of primary liver malignancy with unique clinical, histological, and biological characteristics. It was first reported as a separate entity by Hugh Edmondson in 1956, who described the typical gross and microscopic features of this neoplasm as “a liver cell carcinoma in which stroma is profuse and the similarity of the cancer cells to normal cells is striking” [1]. Since then, it has been further studied, and its diagnostic criteria have been clearly established, the tumor express characteristics of conventional hepatocellular carcinoma (HCC) including cytokeratin 7 a cholangiocellular marker; however studies suggest that no consistent mutation is known in FLC so far [2].

FLC accounts for up to 8% of all HCC, occurs predominantly in younger individuals, has no gender predilection, and develops in the absence of underlying liver disease [3–5]. It is considered to have a better prognosis, due to the fact that it is typically present in noncirrhotic patients with no other comorbid conditions [5]. In contrast to this distinct form of liver cancer, classical HCC represents the most common histologic subtype, occurring mainly in older men with underlying cirrhosis.

To our knowledge, only three cases of a liver carcinoma with elements of both classical HCC and FLC intimately mixed within one mass have been reported [6, 7]. Two other cases describe synchronous tumors [5, 8, 9]. Both the molecular background and the pathogenesis of FLC are obscure and limited, possibly owing to the relatively low number of cases studied by individual groups [10]. Herein, we present a new case of a mixed FLC and classical HCC.

## 2. Case Report

A 37-year-old female presented to our clinic with a two-month history of diffuse abdominal discomfort and epigastric pain. The abdominal ultrasound showed a 9.6 cm oval-shaped, well-demarcated, thin-walled, heterogeneous mass located in the left lateral segment of the liver (segments II and III). CT and MRI scans revealed a well-defined, encapsulated, nonenhancing mass with a central scar, surrounded by normal liver parenchyma. The radiologic diagnosis suggested a FLC in a noncirrhotic liver. Laboratory tests showed abnormally elevated transaminases. Serum tumor markers, including a-fetoprotein (AFP) and carcinoembryonic antigen (CEA), were within normal limits. Viral hepatitis and autoimmune markers were negative. A biopsy was performed at an outside institution before referral, and a diagnosis of classical HCC with a trabecular pattern in a noncirrhotic liver was rendered. The patient's past medical history was unremarkable, and she had no risk factors for chronic liver disease. Physical examination revealed a painful mass that comprised the upper abdomen.

**Figure 1:**
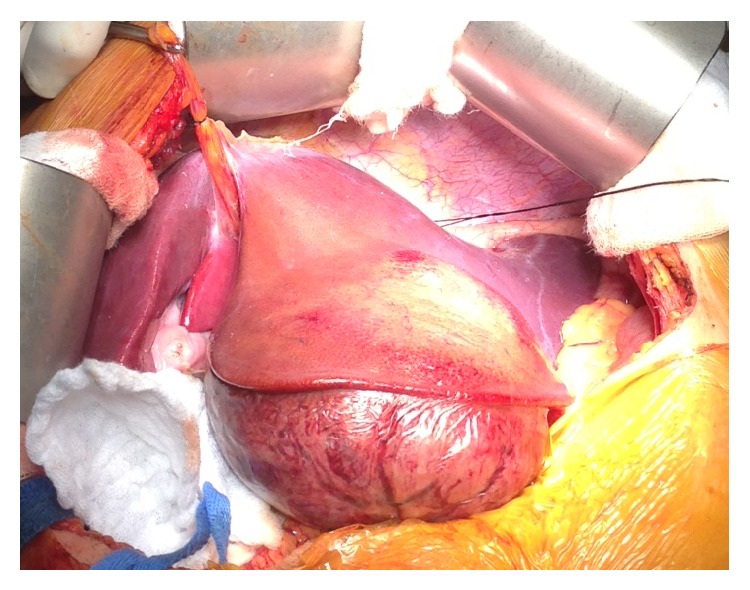
Surgical site showing tumor located in the left-lateral hepatic lobe next to grossly normal liver parechyma.

The patient underwent an uneventful open left-lateral hepatectomy (Figure 1) and was discharged home two days after. Five months after surgery, the patient is alive with no clinical or radiological signs of recurrence.

## 3. Materials and Methods

Macroscopic examination of the resected specimen revealed a multinodular, well-circumscribed, and partially encapsulated mass that measured 13 cm in greatest dimension. Cut surface showed a variegated appearance with some yellow discoloration and central necrosis. The surrounding liver parenchyma had a normal appearance and no evidence of fibrosis or nodularity was seen. The mass was located 3 cm from the surgical margin, and no other lesions were noted.

## 4. Microscopic Examination

Microscopically, the tumor had two different cell populations, one of which showed large, polygonal cells with eosinophilic cytoplasm, large nuclei, prominent nucleoli, and rare mitoses. In addition, there were scattered cytoplasmic inclusions with a ground-glass appearance, known as pale bodies. The tumor cells were surrounded by a fibrolamellar type stroma. These findings were consistent with FLC.

**Figure 2:**
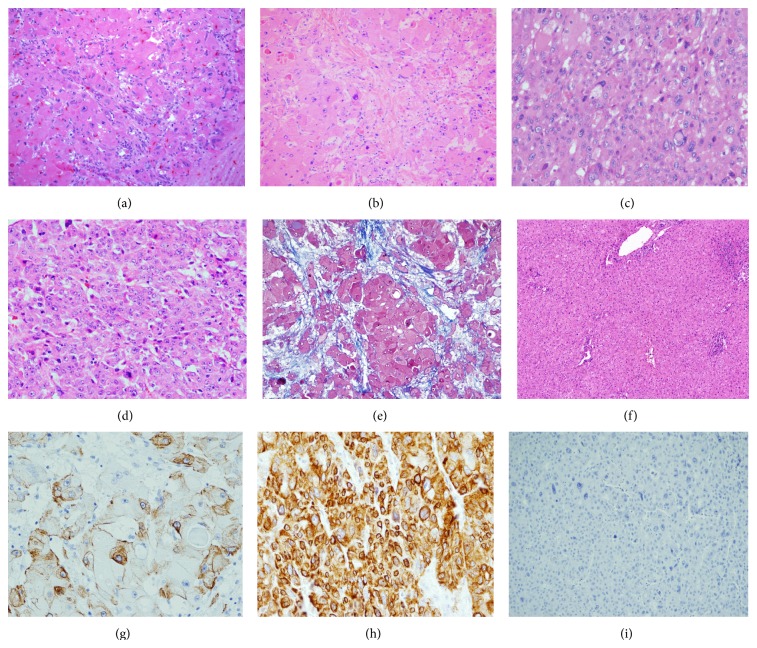
(a) (H and E stain, 200x) Mixed tumor composed of two different components, one with large, polygonal cells with eosinophilic cytoplasm, consistent with FLC (top left). Adjacent to it without transition (bottom right), the second component shows a neoplastic proliferation of hepatocytes with high N : C ratio and a trabecular pattern compatible with classical HCC. (b) (H and E stain, 200x) typical abundant lamellar connective tissue characterizing FLC. (c) (H and E stain, 400x) High power view of FLC component showing large neoplastic cells with abundant eosinophilic cytoplasm and pale bodies. (d) (H and E stain, 200x) Photograph of classical HCC showing nests of neoplastic hepatocytes with abnormal architecture showing thick liver plates and endothelial wraping. (e) (Masson's trichrome stain, 200x) Lamellar connective tissue in the FLC component. (f) (H and E stain, 100x) Low power view of adjacent liver parenchyma withouth fibrosis and few foci of nonspecific lymphocytic parenchymal inflammation with retained architecture. (g) (CK7-200x) Immunohistochemistry for CK7 showing positivity in FLC and (h) (CK7-400X) in the HCC. (i) (CK19-400X) Negative in HCC.

Adjacent to these areas, a second group of cells with marked cytologic atypia, nuclear pleomorphism, hyperchromatic nuclei, intracytoplasmic Mallory's hyaline and eosinophilic globules, and zones of necrosis was observed. Morphologically, these areas were consistent with classical HCC with a trabecular pattern. Vascular invasion and tumor thrombi were also present. The background liver showed no evidence of cirrhosis (Figure 2).

Immunohistochemical studies showed both components to be reactive for hepatocyte (Hepar-1) and to focally express AFP. Endothelial wrapping was highlighted with CD34, and a canalicular staining pattern was observed with polyclonal CEA. Both groups of neoplastic cells were positive for CK7 and negative for CK19. A diagnosis of a hepatocellular carcinoma with both FLC and classical HCC features was rendered.

## 5. Discussion

We present a case of a liver tumor with unusual morphology and features of both classical HCC and FLC in a patient with no predisposing factors. FLC is a malignant hepatocellular tumor with distinct clinical and pathologic characteristics that differ from classical HCC, predominantly in FLC there is no consistent mutation, and shows fewer chromosomal abnormalities compared with HCC [2].

To our knowledge, there are only five cases reporting the presence of classical HCC coexistent with FLC in the literature (Table 1). Two cases described two synchronous tumors of the liver. In the first case, one mass was located in the caudate lobe with FLC elements, and the other with classical HCC features located in the lateral segment [8]. The second case had two contiguous masses in the right lobe that were separated by a rim of fibrous tissue that surrounded the classical HCC component, which was the largest tumor. The smaller mass represented the FLC [9]. As in our case, there are only three previous reports that describe a single hepatic mass composed of the two mixed histologic subtypes [5–7].

FLCs have a predilection for the left lobe of the liver, and grossly, they tend to be yellow to pale tan and firm [3, 11, 12]. The average size ranges from 9 to 14 cm in greatest dimension, and a central scar may be found in up to 75% of cases [3]. Occasionally, these tumors can contain small foci of necrosis, hemorrhage, and calcifications [3, 13].

Microscopically, the defining features of FLCs are large polygonal cells with abundant eosinophilic (oncocytic) cytoplasm, large vesicular nuclei, large nucleoli, and lamellar type fibrosis [3, 11, 12]. Calcifications and round cytoplasmic inclusions (pale bodies) are seen in up to 50% of the cases [11]. Characteristically FLCs are strongly positive for CK7, and a recent study found that positivity for CD68 is highly sensitive [3, 14].

On the other hand, classical HCC is usually nodular with or without a fibrous capsule, and it is softer than the normal surrounding liver. It varies in color and can be yellow, green, brown, or heterogeneous [11].

Histologically, the main hallmark of classical HCC is its similarity to the normal liver both in its plate-like growth and its cytology [13]. It is characterized by plates of hepatocytes with eosinophilic or clear cytoplasm that are separated by sinusoid-like spaces lined by a single layer of endothelial cells that wrap around the plates of hepatocytes [11]. Architecturally, the cells are often arranged in trabecular, acinar/pseudoglandular, or solid/compact patterns [12]. Malignant hepatocytes are often pleomorphic, and cytoplasmic accumulations are commonly present, including Mallory's hyaline, bile, steatosis, iron, copper, and eosinophilic globules containing proteins such as a-1-antitrypsin [12].

Immunohistochemically, classical HCC is characterized by cytoplasmic positivity for Hepar-1 (approximately 90% of the cases), patchy AFP, fibrinogen, and cytokeratins 8 and 18. Polyclonal CEA and CD10 show canalicular patterns of positivity. Special stain for reticulin shows patchy or diffuse loss of the reticulin network [11]. CD34 highlights the endothelial wrapping. Immunostains for Glypican 3, heat shock protein, and glutamine synthetase are also positive in HCC. Usually, classical HCC is negative for CK7 (≥70% of cases).

In the present case, the patient had no evidence of underlying liver disease, she had no risk factors for HCC, AFP levels were within normal limits, HBV titers were negative, and imaging showed a mass in the left lobe of the liver, all of which are characteristic clinical features of FLC. In contrast, she presented with vague, nonspecific abdominal signs and symptoms, and with mild elevation of the liver function tests, which may be seen in both types of tumors. The age at presentation of the disease (37 years old) is not usual for either classical HCC or FLC.

Microscopically, the tumor showed distinct areas in which the morphological elements of classical HCC and FLC were mixed. However, the immunoreactivity for CK7, which was expected to be expressed solely by the FLC component, was positive in both groups of neoplastic cells. It should be noted that some (≤30%) classical HCCs may be reactive for CK7, in particular those in children and young adults [3, 15]. In this sense, the DNAJB1-PRKACA chimeric transcript that is missed in the domain that binds the regulatory subunits of PKA, may represent a diagnostic marker in FLC, nonetheless further research has to be done to define it as a therapeutic target [15].

Currently there are no molecular diagnostic tests for FLC; previous studies detected cAMP-dependent protein kinase A (PKA) in peripheral blood as a universal serum biomarker for cancers of various cell types [16]. The* DNAJB1-PRKACA* chimeric transcript that is missed in the domain that binds the regulatory subunits of PKA and may represent a diagnostic marker in FLC; nonetheless further research has to be done to define it as a therapeutic target [17].

We did not find any risk factors or environmental exposure in the patient's history that could explain the etiology of this neoplasm. As a matter of fact the surrounding liver parenchyma was normal and did not have any signs of cirrhosis or underlying liver disease.

In summary, we hope that our report will increase awareness of this combined entity in the liver and that further experience contributes in the establishment of prompt and accurate diagnoses involving not only proteins expression through immunohistochemistry but also genetic studies in future cases.

## Figures and Tables

**Table 1:** Summary of clinical findings of reported cases of coexistent FLC and classical HCC.

Author	Age (years)	Sex	Clinical presentation	Tumor size and lobe	Liver enzymes	AFP	CEA	HBV antigen titers	Non- neoplastic liver	Other findings	Type of tumor
Okada et al. [8]	56	M	Referral	1.9 cm left and 1.8 cm caudate	Elevated	Elevated	WNL	Negative	Cirrhosis	Leukopenia	Synchronous FLC and HCC
Singh and Ramakrishna [9]	14	M	Abdominal pain	10 cm right and 8 cm right	Elevated	WNL	WNL	Negative	None present	None	Synchronous FLC and HCC
Seitz et al. [5]	27	F	Epigastric pain	16 cm right	Elevated	WNL	NA	Negative	None present	None	Mixed FLC and HCC
Reuland et al. [6]	39	F	Incidental finding	NA	WNL	WNL	WNL	NA	None present	Leukocytosis and elevated ESR	Mixed FLC and HCC
Okano et al. [7]	52	M	Incidental finding	3.5 cm left	WNL	Elevated	WNL	Negative	None present	None	Mixed FLC and HCC
Castro-Villabón et al. (present case)	37	F	Abdominal distension and epigastric mass	13 cm left	Elevated	WNL	WNL	Negative	None present	None	Mixed FLC and HCC

AFP: a-fetoprotein; CEA: carcinoembryonic antigen; HBV: hepatitis B virus; ESR: erythrocyte sedimentation rate; WNL: within normal limits; NA: not available.
